# Spatio-Temporal Changes of Lymphatic Contractility and Drainage Patterns following Lymphadenectomy in Mice

**DOI:** 10.1371/journal.pone.0106034

**Published:** 2014-08-29

**Authors:** Sunkuk Kwon, Germaine D. Agollah, Grace Wu, Eva M. Sevick-Muraca

**Affiliations:** 1 Center for Molecular Imaging, The Brown Foundation Institute of Molecular Medicine, The University of Texas Health Science Center, Houston, Texas, United States of America; 2 The University of Texas Graduate School of Biomedical Sciences at Houston, The University of Texas MD Anderson Cancer Center, Houston, Texas, United States of America; Okayama University, Japan

## Abstract

**Objective:**

To investigate the redirection of lymphatic drainage post-lymphadenectomy using non-invasive near-infrared fluorescence (NIRF) imaging, and to subsequently assess impact on metastasis.

**Background:**

Cancer-acquired lymphedema arises from dysfunctional fluid transport after lymphadenectomy performed for staging and to disrupt drainage pathways for regional control of disease. However, little is known about the normal regenerative processes of the lymphatics in response to lymphadenectomy and how these responses can be accelerated, delayed, or can impact metastasis.

**Methods:**

Changes in lymphatic “pumping” function and drainage patterns were non-invasively and longitudinally imaged using NIRF lymphatic imaging after popliteal lymphadenectomy in mice. In a cohort of mice, B16F10 melanoma was inoculated on the dorsal aspect of the paw 27 days after lymphadenectomy to assess how drainage patterns affect metastasis.

**Results:**

NIRF imaging demonstrates that, although lymphatic function and drainage patterns change significantly in early response to popliteal lymph node (PLN) removal in mice, these changes are transient and regress dramatically due to a high regenerative capacity of the lymphatics and co-opting of collateral lymphatic pathways around the site of obstruction. Metastases followed the pattern of collateral pathways and could be detected proximal to the site of lymphadenectomy.

**Conclusions:**

Both lymphatic vessel regeneration and co-opting of contralateral vessels occur following lymphadenectomy, with contractile function restored within 13 days, providing a basis for preclinical and clinical investigations to hasten lymphatic repair and restore contractile lymphatic function after surgery to prevent cancer-acquired lymphedema. Patterns of cancer metastasis after lymphadenectomy were altered, consistent with patterns of re-directed lymphatic drainage.

## Introduction

Lymphedema is a disfiguring and debilitating condition of impaired lymph uptake and/or transport manifested by chronic swelling, tissue fibrosis, and reduced immune response [1]. Unlike primary lymphedema, which is a rare inherited disorder resulting from malformations or abnormalities of the lymphatic system, secondary lymphedema is an acquired lymphatic disorder resulting from trauma, infection, or cancer treatment such as surgery/radiation that damages the lymphatic system [1]. The onset of secondary lymphedema can occur within a few weeks up to years following cancer treatment and strikes up to 10–85% of the growing population of cancer survivors, depending on the cancer type [2], [3]. Recent developments, including the discovery of key molecular regulators of lymphatic development and vessel growth, and identification of casual genes contributing to primary lymphedema, have enabled a better understanding of the lymphatic system [4], [5]. However, the standard-of-care treatment for the more common cancer-acquired lymphedema has remained largely unchanged for the past 80 years, and because there is no means to predict which cancer survivors will encounter the disease, there have been no prophylactic therapies to prevent the onset of this chronic, incurable condition. Current treatment options are limited to repetitive physiotherapeutic interventions that include manual lymph drainage, compression bandaging, and pneumatic compression, in order to develop a collateral pathway from the affected limb toward the normal lymphatic system and stimulate existing lymphatics [1], [6].

In order to evaluate the etiology of lymphedema, several animal models of acquired lymphedema in the limb of dogs [7], [8] and rats [9]–[11], rabbit ear models [12]–[14], and mouse tail models [15]–[17] have been developed via disruption of normal lymphatic transport by surgically removing lymph nodes (LNs) and/or significant tissue mass. Because lymphadenectomy is perhaps most relevant to post-surgical cancer-related lymphedema, therapeutic strategies to stimulate lymphatic vessel regrowth that include LN transplant, *Vegf-c* gene therapy and/or other pharmacological treatments have been proposed for amelioration of acquired lymphedema [18]–[24]. When a LN is excised, pre- and post-nodal collecting lymphatic vessels are also damaged. Thus, normal lymphatic drainage is blocked, and stagnated lymphatic fluid must initially be rerouted around the site of LN removal through collateral lymphatics. In addition, others have suggested that there is spontaneous reconnection of afferent and efferent collecting lymphatic vessels after lymphadenectomy [18]–[20], [23], [25]. The initial, surgically induced acute experimental edema eventually regresses naturally, due to both the regenerative capacity of the lymphatics and to the development of collateral pathways around the site of obstruction. In addition, evidence from near-infrared fluorescence (NIRF) lymphatic imaging in normal controls and lymphedema subjects suggest that there is impaired lymphatic “pumping” function even in early stages of the condition [26], suggesting that normal regenerative processes involve functional restoration of lymphatic “pumping”. Yet, little is known about the normal regenerative processes of the lymphatics in response to lymphadenectomy and how these responses can be accelerated, delayed, or how they impact subsequent cancer metastasis. Longitudinal, non-invasive imaging of lymphatic functional and architectural changes provides a window for assessing the role of the lymphatics in repair processes and consequently for choosing efficacious treatment approaches based upon direct evidence of improved lymphatic function. In this study, we noninvasively imaged changes in lymphatic contractile function and drainage pathways for a period of up to 4 weeks after popliteal LN (PLN) dissection in mice using NIRF lymphatic imaging.

## Materials and Methods

### Ethics statement

Animals were maintained in a pathogen-free mouse facility accredited by the American Association for Laboratory Animal Care (AALAC). All experiments were performed in accordance with the guidelines of the Institutional Animal Care and Use Committee (IACUC). Animal experiments were approved by University of Texas Health Science Center Animal Welfare Committee (AWC).

### Experimental animal models

**Figure 1:**
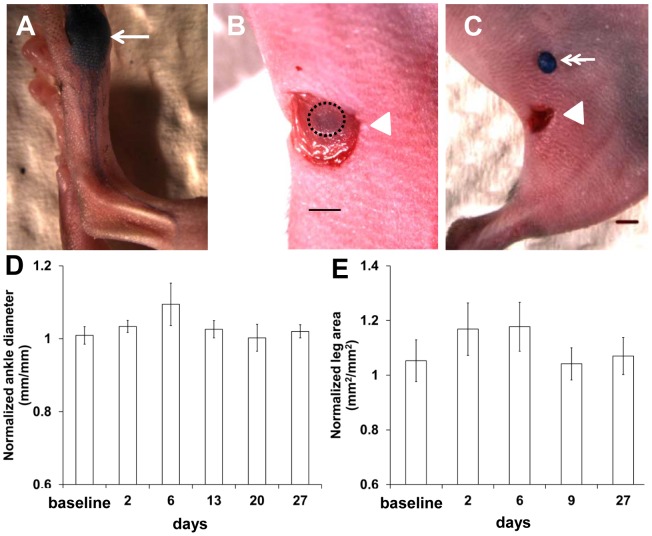
Representative intravital color image of the mouse foot showing lymphatic trafficking of EBD 5 mins after i.d. injection of EBD (A). Arrow, EBD injection site. After a small incision of the skin, the blue PLN under fascia (black dotted circle) was observed (B). Arrowhead, surgical incision site. Scale, 1 mm. The EBD-filled PLN was excised (C). Double arrow, dissected PLN. Scale, 1 mm. Ankle thickness (D) and cross-sectional leg area (E) from axial CT slices were measured from stereoscopic imaging and CT scans, respectively, and the operated side was normalized to the contralateral right side.

Eight to ten week-old female C57BL/6 mice (Charles River, Wilmington, MA) were housed and fed sterilized pelleted food and sterilized water. Mice were anesthetized with isoflurane (2% oxygen) and maintained on a warming pad at 37°C. Two µl of 1% Evans blue dye (EBD) solution (5 mg/ml; Sigma) was injected into the dorsal aspect of the left paw and taken up by the lymphatics (See Figure 1A). Bupivacaine (0.25%; 1 mg/kg) was given subcutaneously and chlorhexidine was applied around the popliteal region. After a small (∼5 mm) incision was made with sharp dissecting scissors through the skin, the blue PLN under fascia was visible (Figure 1B). Under a stereomicroscope (MZ16 A, Leica Microsystems, Inc.) the fascia on top of the blue PLN was pinched and pulled with a forceps and carefully incised with dissecting scissors. Subsequently, the blue PLN was carefully removed with minimal disruption to the surrounding tissues. Following PLN removal, wounds were closed with surgical glue (3M Vetbond). Buprenorphine (0.1 mg/kg) was administered intraperitoneally once after surgery.

### B16F10 tumor model

B16F10 murine melanoma cells were obtained from American Type Culture Collection (ATCC) and cultured in DMEM-F12 (GIBCO) supplemented with 10% FBS. B16F10 cells (5×10^5^ cells per mouse in 10 µl sterile PBS) were then implanted intradermally in the dorsal aspect of the left paw of mice (n = 4) which had undergone surgical removal of the left PLN 27 days prior. Mice (n = 4) without lymphadenectomy were also injected with B16F10 cells. Three weeks after inoculation, mice were euthanized, and intravital imaging using a stereoscope was performed to image LN metastasis. After intravital imaging, tissues were collected for histology.

### Fluorescence lymphatic imaging and computed tomography

Animals were clipped and residual hair removed with depilatory cream (Nair, Church & Dwight Co., Inc.) 24 hrs before imaging. Mice were imaged for baseline characteristics prior to PLN removal and for up to 4 weeks after surgery. Anesthetized mice with isoflurane were maintained on a warming pad. A volume of 2 µl of 645 µM of indocyanine green (ICG; Akorn, Inc.) dissolved in a mixture of distilled water and 0.9% sodium chloride in a volume ratio of 1∶9 was injected intradermally to the dorsal aspect of the left foot using 34-gauge needles (Nanofil, World Precision Instruments, Inc.). Fluorescence images were acquired immediately after and for up to 10 min after i.d. injection using a custom-built NIRF imaging system as described previously [27], [28]. To achieve a greater magnification, a macrolens (Infinity K2/SC video lens, Edmund Optics Inc.) was used. At the last imaging session, the skin above the left PLN was removed for intravital color images after i.d. injection of EBD.

Lymphatic drainage through thoracic duct from the lumbar LNs (LLNs) was imaged in another two groups of mice: (i) mice without lymphadenectomy and (ii) two days following lymphadenectomy. Invasive, far-red and NIRF imaging was conducted 5 mins after i.d. injection of 2 µl of Alexa680-bovine serum albumin (BSA) and 2 µl of ICG to the dorsal aspect of the right and left hind paw, respectively. The peritoneal and thoracic cavities were exposed and imaged immediately after animals were euthanized.

*In vivo* small animal CT imaging was performed to measure arm area from transverse CT slices [29] using an INVEON multimodality CT (SiemensMedical Solutions, Knoxville, TN). CT parameters were set at 80 kV, 500 mA, 290 ms exposure time at each of the 120 rotation steps over 220° at medium-low system magnification. CT images were reconstructed using a Feldkamp cone-beam algorithm. Image analyses from CT were performed using Inveon Research Workplace software (Siemens Medical Solutions). Cross-sectional area between tarsocrural and distal tibiofibular joints in the left leg was measured by analysis of axial CT slices and normalized to that in the contralateral right leg. Ankle diameter of hind limbs was also measured using a stereomicroscope. The ankle diameter of the left side (side of lymphadenectomy) was normalized to the contralateral right ankle diameter.

### Analysis of lymphatic vessel function

Matlab (The MathWorks, Inc., Natick, MA) and ImageJ (National Institutes of Health, Washington, DC) were used to analyze the fluorescence imaging data in the following manner. To assess lymphatic contractility, fixed regions of interest (ROIs) in fluorescent afferent and efferent lymph channels were defined on fluorescence images. The mean of the fluorescence intensity within each ROI in each fluorescence image was then calculated and plotted as a function of imaging time as previously described [27].

### Immunohistochemical (IHC) analysis and whole-mount staining

Skin samples were fixed in 10% formalin overnight before transfer into 70% ethanol and embedded in paraffin. 4 µm sections were used in all staining procedures which included hematoxylin-eosin (H&E). For LYVE-1 detection, following paraffin removal and antigen retrieval using citrate buffer pH 6, tissues were blocked with 5% BSA and stained with rabbit anti-mouse LYVE-1 antibody (1∶200; AngioBio) and biotin-anti rabbit secondary antibody (1∶30; Vector Labs). VectaStain Elite ABC peroxidase detection system and DAB chromagen were used before tissues were counter-stained with hematoxylin (Vector Labs). LYVE-1 expression in three different fields in each section was examined at x40 magnification (Leica Microsystems Inc.). Image-Pro Plus (Media Cybernetics) software was used to analyze the number of lymphatic vessels, and the percentage of positively stained lymphatic vessels per area was determined.

For whole mount staining [30], tissues were fixed in 4% paraformaldehyde (PFA) overnight at 4°C. Adipose tissue was removed under a dissecting microscope and tissues washed with 0.3% TritonX-PBS at least 10 times over 4 hours. Samples were blocked with 0.2% BSA, 5% goat serum in 0.3% TritonX-PBS for 2 hours at room temperature before staining with LYVE1 antibody (1∶100; Angiobio) at 4°C overnight with shaking. Tissues were washed for at least 6 hours with 0.3% TritonX-PBS before being incubated at 4°C overnight with secondary antibody (Alexa-Flour 488 nm; goat anti-rabbit; 1∶500 in 0.3% TritonX-PBS) with shaking. Tissues were washed at least 10 times over 6 hours with 0.3% TritonX-PBS before 10 min fixation with 4% PFA. Final wash was performed with PBS 5 times over 30 mins before tissues were mounted with Vectashield (Vector Labs). LYVE-1 expression in at least three different fields in each section was examined at x20 magnification (Leica Microsystems Inc.). Quantification of lymphatic vessel area fraction was performed using ImageJ (NIH).

### Statistical analysis

Data were presented as average values ± standard error. Statistical analysis was performed with Prism 5 (Graphpad Software, Inc). The D'Agostino and Pearson omnibus tests were used to assess data normality. Changes of ankle diameter, leg area, and lymphatic contraction frequency were analyzed with one-way repeated-measures analysis of variance (ANOVA) with a Bonferroni posttest (parametric) or a Friedman test with a Dunn posttest (nonparametric) for multiple comparisons over time. A one-way ANOVA with Bonferroni post hoc was used when three or more groups were compared. The student t-test was also used for comparisons made between two groups. The differences were considered significant at p<0.05.

## Results

### Altered lymphatic drainage after PLN removal

**Figure 2:**
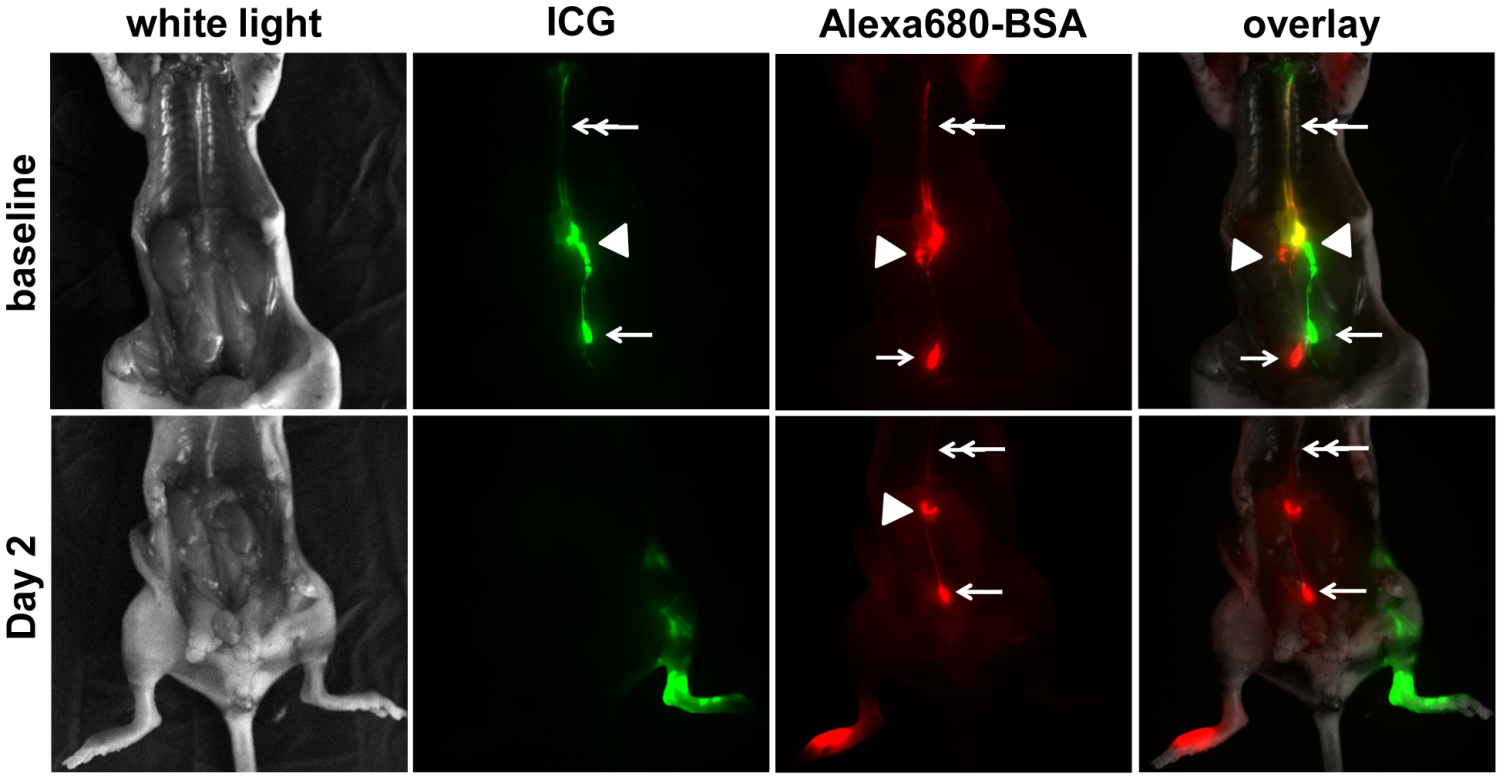
Representative intravital fluorescence images of mice 5 mins after i.d. injection of ICG (green) and Alexa680-BSA (red) on the dorsal aspect of left and right paw, respectively, in control mice prior to surgery and 2 days after surgical removal of the left PLN. Double arrow, thoracic duct. Arrowhead, RLN. Arrow, LLN.

After i.d. injection of EBD on the dorsal aspect of the paw (Figure 1A; arrow depicting EBD injection site), blue dye-stained popliteal afferent lymphatic vessels were visualized. In addition, the left blue PLN under fascia (black dotted circle in Figure 1B) was observed following a small incision in the skin over the PLN and removed through minimally invasive surgery as shown in Figure 1C. Image analysis from stereoscopic imaging (Figure 1D) and CT scans (Figure 1E) showed mild swelling of the operated hind leg for up to 6 days post-surgery and a decrease in swelling thereafter, demonstrating that minimally invasive PLN dissection did not produce acute lymphedema. Because ICG administered to the dorsal aspect of the paw drains to the PLN, then the ischial LNs (IsLNs) and LLNs, into renal LNs (RLNs), and finally to the thoracic duct along the midline, we imaged mice after i.d. injection of 2 µl of Alexa680-BSA and 2 µl of ICG to the dorsal aspect of the right and left hind paws, respectively. Figure 2 demonstrates that control mice without lymphadenectomy showed normal lymphatic trafficking of (i) ICG from the left LLN (arrow) to the left RLN (arrowhead) and finally to the thoracic duct (double arrow) and (ii) Alexa680-BSA from the right LLN (arrow) to the right RLN (arrowhead) and finally to the thoracic duct (double arrow). However, 2 days after lymphadenectomy, mice exhibited lymphatic drainage only from the non-operated right side where Alexa680-BSA was injected. ICG-laden lymph did not drain through the thoracic duct due to blockage of normal lymph flow resulting from surgical removal of the left PLN.

**Figure 3:**
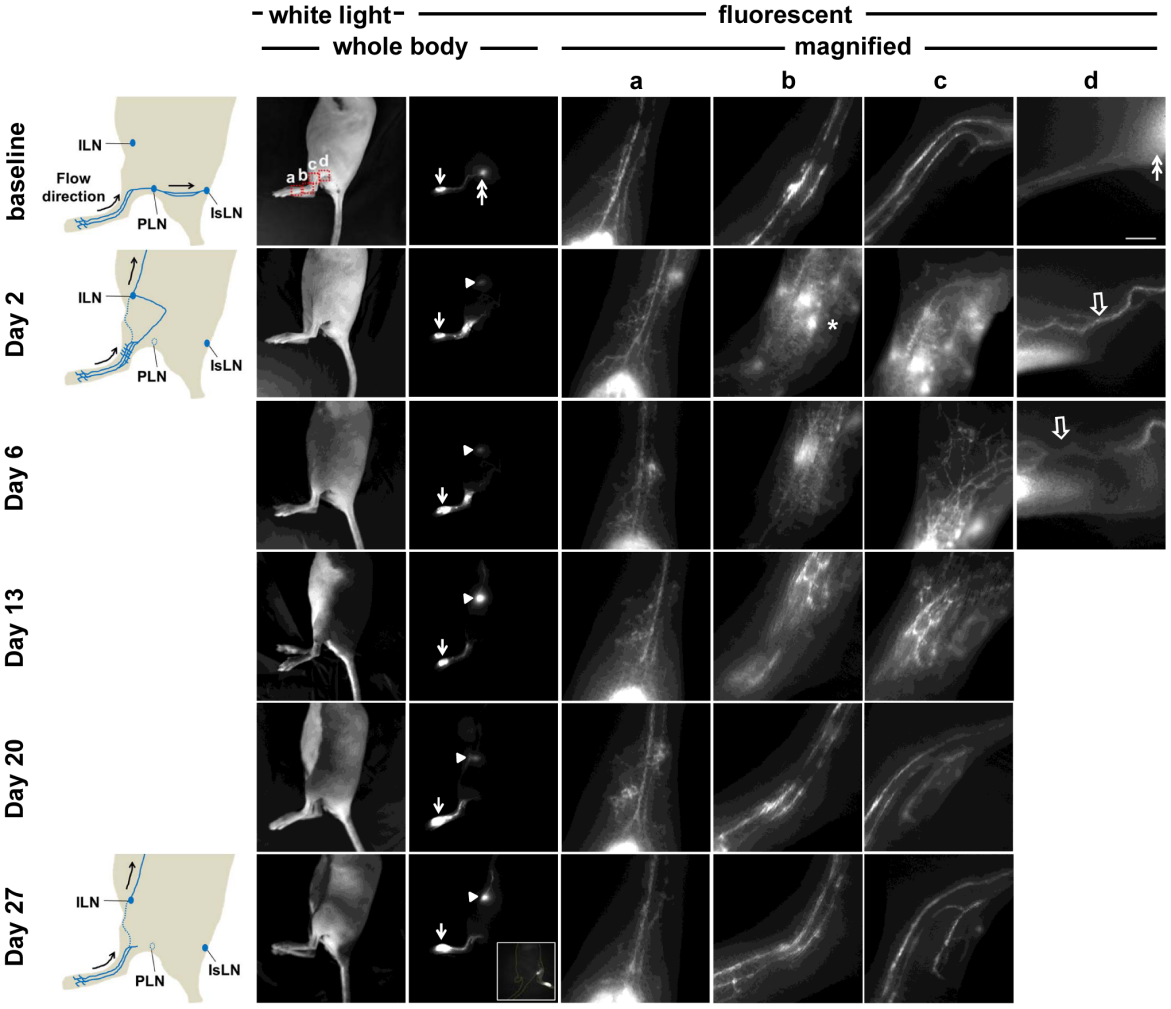
White light and fluorescent images in mice prior to and 2, 6, 13, 20, and 27 days after PLN removal. Images were acquired 10 mins after i.d. injection of ICG. Magnified fluorescent images of the red rectangles (a, b, c, and d) were also acquired. Arrow, ICG injection site. Double arrow, PLN. Arrowhead, ILN. Asterisk, bright spots due to leaked-out ICG. Open arrow, newly detected fluorescent lymphatic vessels after PLN surgery. Inset, fluorescent image in the ventral view of the mouse showing lymphatic drainage to the ILN. Scale bar, 1 mm.

Although EBD is suitable for intravital imaging of lymphatic vessels and LNs in dissected tissues, it is difficult to longitudinally image the distribution of EBD in mice *in vivo*. Therefore, we performed longitudinal NIRF imaging to investigate changes of lymphatic drainage patterns in mice after PLN removal. As shown in Figure 3, 16 out of 19 mice showed well defined lymphatic vessels draining from the injection site on the paw to the PLN and further drainage to the IsLN prior to lymphadenectomy. The 3 out of 19 mice with different drainage patterns are described further below. The specific drainage pattern in each mouse was consistently observed in two baseline imaging sessions prior to lymphadenectomy.

Longitudinal NIRF lymphatic imaging demonstrated transient changes of lymphatic drainage patterns after PLN removal (Figure 3). At 2 days post-surgery, fluorescence-laden lymph, which previously drained from the injection site to PLN at baseline, was rerouted and alternatively drained to the inguinal LN (ILN). This redirection of lymphatic drainage was observed in all 16 mice. Magnified fluorescent images using a macrolens showed that ICG dye pooled proximally at the wound site and was not transported across the site of LN removal through the afferent lymphatic vessel to the PLN (Figure 3). We also observed substantial dermal backflow through dermal capillaries and extravascular ICG as evidenced by bright spots, indicating impairment of normal lymphatic drainage of the ICG-injected paw 2 days after lymphadenectomy. At 6 days post-surgery, fewer fluorescent spots were observed, although dermal backflow due to abnormal filling of the lymphatic capillaries remained. Disrupted lymphatic drainage observed as extravascular ICG and dermal backflow started to disappear beginning on day 13 post-surgery. Eventually, similar patterns of baseline lymphatic drainage through well-defined lymphatic vessels draining from the injection site were detected at 20 days post-surgery, as shown in Figure 3. However, lymphatic drainage pathways draining to the IsLN were not detected, as seen in baseline imaging. Instead, inguinal afferent lymphatic vessels draining the dye anteriorly to the ILN were observed, as shown in the inset in Figure 3.

**Figure 4:**
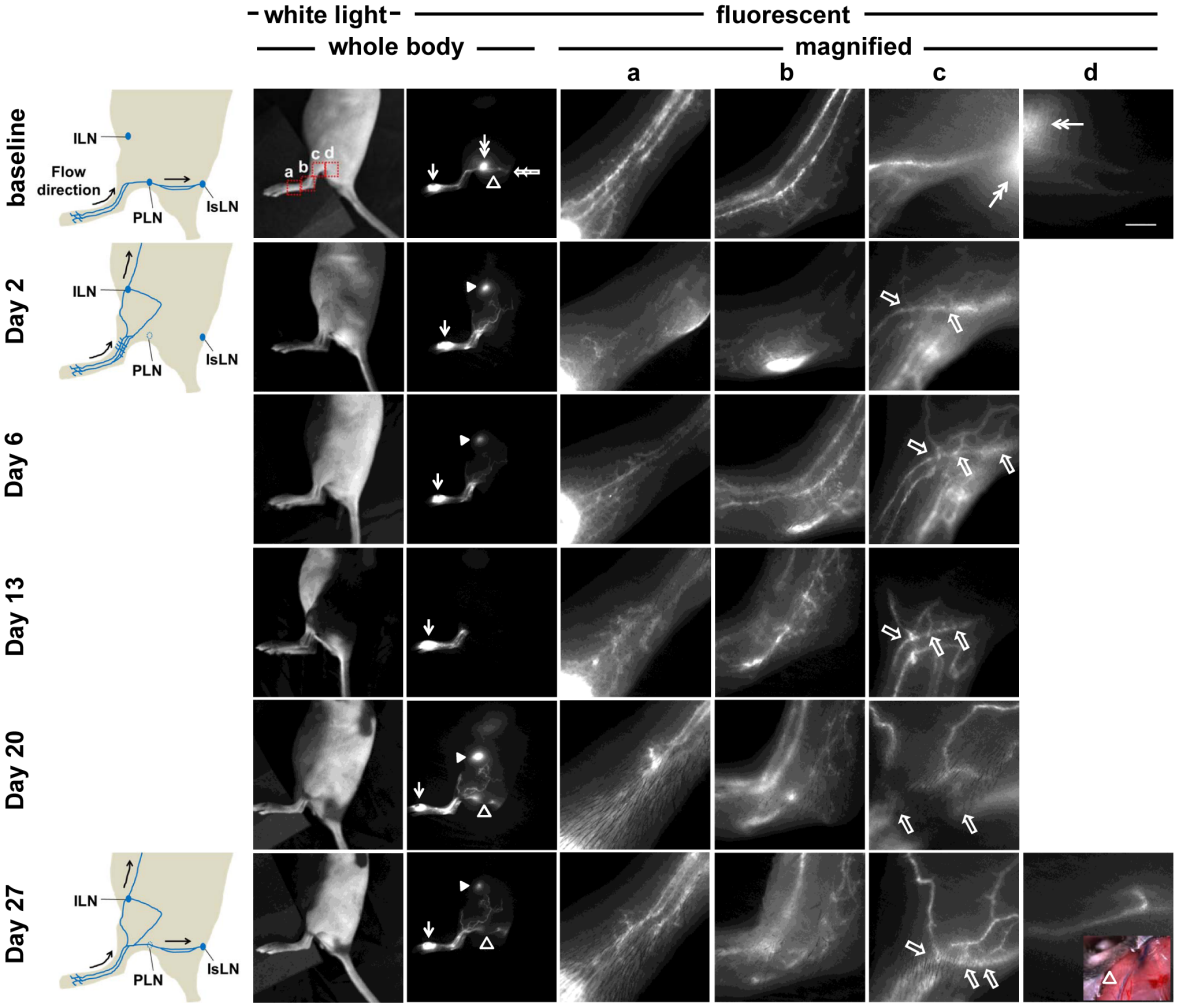
White light and fluorescent images in mice prior to and 2, 6, 13, 20, and 27 days after PLN removal. Images were acquired 10 mins after i.d. injection of ICG. Magnified fluorescent images of the red rectangles (a, b, c, and d) were also acquired. Arrow, ICG injection site. Double arrow, PLN. Open arrowhead, popliteal efferent collecting lymphatic vessel. Arrowhead, ILN. Open arrow, lymphatic vessel branch points from previously observed lymphatic vessels after PLN surgery. Double open arrow, IsLN. Inset, Intravital color image after i.d. injection of EBD. Scale bar, 1 mm.

Fluorescent lymphatic vessels draining both to the IsLN through the wound site and to the ILN were also observed in mice that underwent lymphadenectomy. As shown in another example in Figure 4, PLN removal also resulted in changes of lymphatic drainage pathways. Two days after the PLN was removed, fluorescent lymphatic vessels were observed to branch off from pre-existing afferent popliteal collecting lymphatic vessels as seen in baseline images (Figure 4). These newly detected functional lymphatic vessels were seen in response to failure of lymph flow across the site of LN removal. These collateral fluorescent vessels in the mouse were observed until 27 days after surgery. In addition, we observed fluorescent internodal collecting lymphatic vessels draining from the site of PLN removal to the IsLN 20 days after surgery, indicative of the restoration of lymphatic continuity and restored lymph transport after lymphangiogenesis in the wound-healing process. The drainage patterns through the internodal collecting lymphatic vessels were similar to those seen in baseline imaging prior to surgery (Figure 4). Intravital color imaging after i.d. injection of EBD also confirmed lymphatic drainage from the injection site to the IsLN through the popliteal region as shown in a color image in the inset in Figure 4.

**Figure 5:**
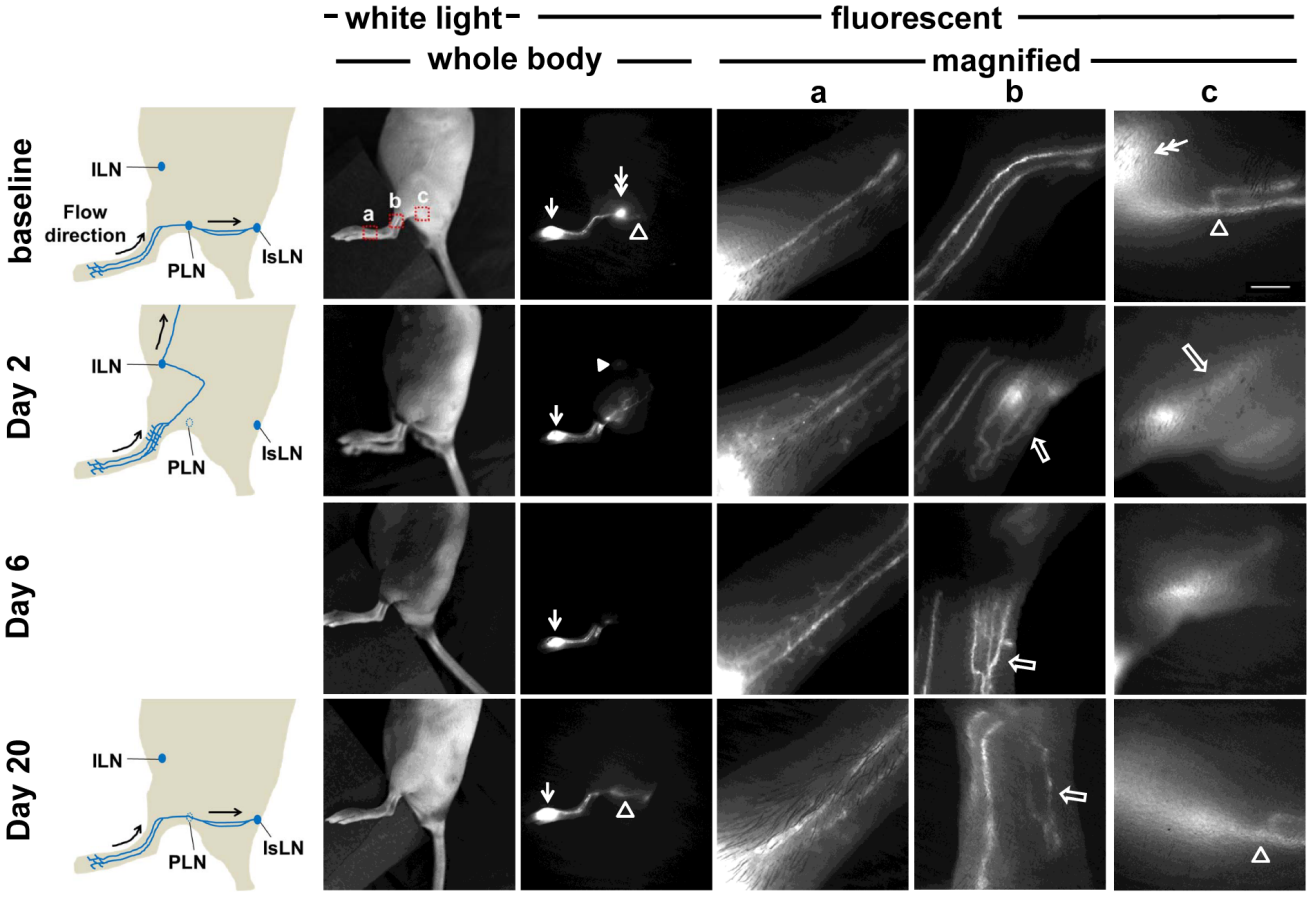
White light and fluorescent images in mice prior to and 2, 6, and 20 days after PLN removal. Images were acquired 10 mins after i.d. injection of ICG. Magnified fluorescent images of the red rectangles (a, b, and c) were also acquired. Arrow, ICG injection site. Double arrow, PLN. Open arrowhead, popliteal efferent collecting lymphatic vessel. Arrowhead, ILN. Open arrow, newly detected fluorescent lymphatic vessels after PLN removal. Scale bar, 1 mm.

We also observed fluorescent collecting lymphatic vessels draining from the injection site to the IsLN, but not to the ILN, within 27 days after the procedure. As shown in Figure 5, at 2 days post-surgery, functional fluorescent lymphatic vessels branching from previously observed pre-existing vessels before surgery were detected. These vessels draining from the injection site to the ILN were not detected at day 20, whereas collecting lymphatic vessels from the injection site to the IsLN through the site of PLN removal were visualized. Thus, alternate drainage pathways were detected due to redirection of lymph flow 2 days after lymphadenectomy and ICG-laden lymph moved within a continuous network of lymphatic vessels due to decreased flow resistance at 20 days post-lymphadenectomy.

**Figure 6:**
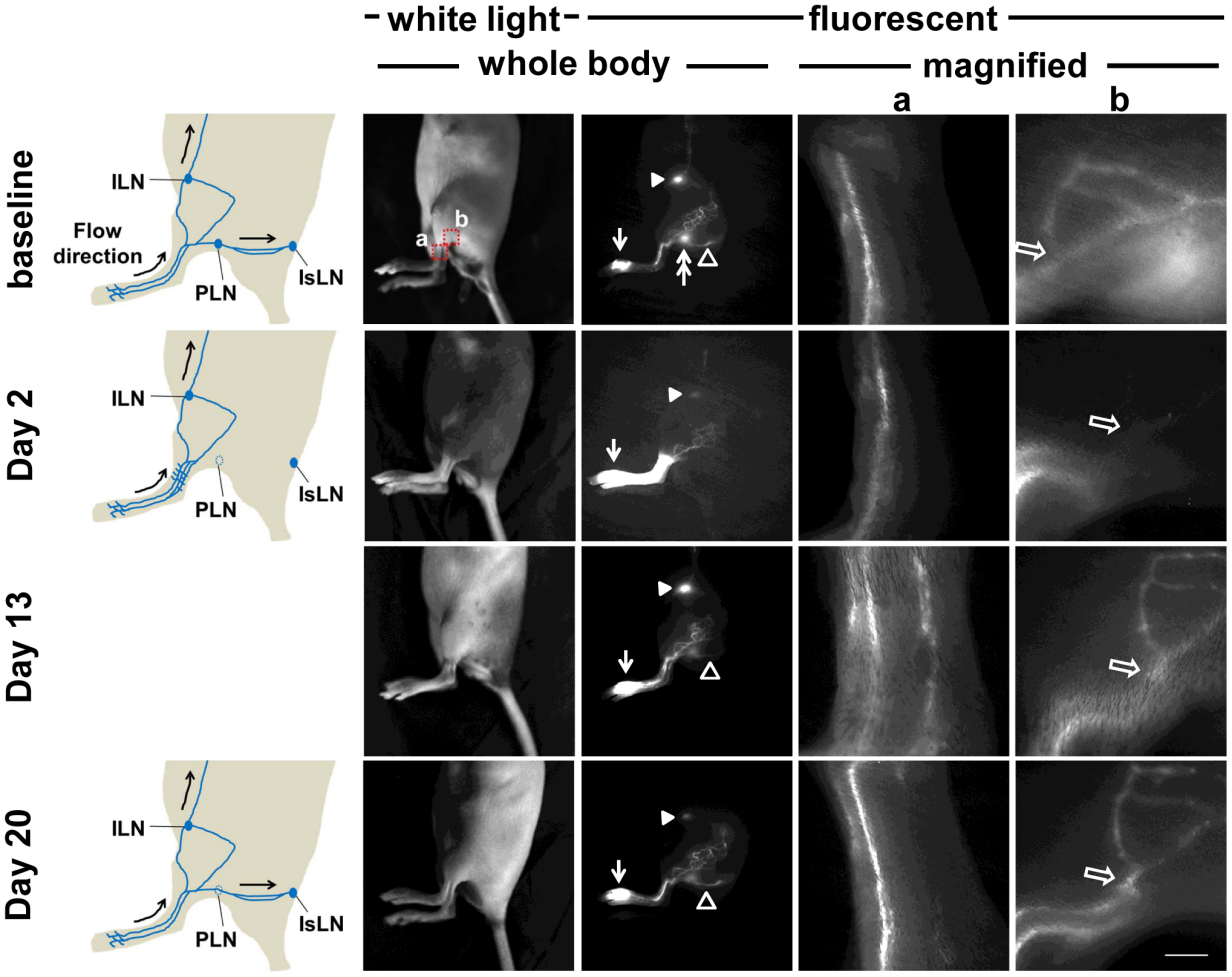
White light and fluorescent images in mice prior to and 2, 13, and 20 days after PLN removal. Images were acquired 10 mins after i.d. injection of ICG. Magnified fluorescent images of the red rectangles (a and b) were also acquired. Arrow, ICG injection site. Double arrow, PLN. Open arrowhead, popliteal efferent collecting lymphatic vessel. Arrowhead, ILN. Open arrow, lymphatic vessels branching off from collecting lymphatic vessels. Scale bar, 1 mm.

As described previously, 3 out of 19 mice showed altered lymphatic drainage pathways at baseline. We detected fluorescent lymphatic vessels draining from the injection site to the IsLN through the PLN as well as to the ILN in these 3 mice (Figure 6). Our longitudinal NIRF imaging demonstrated that at 2 days post-surgery, lymphatic drainage patterns in the hind limb were similar to those observed prior to surgery except there was no drainage to the site of PLN removal. At 13 days after surgery, we detected functional collecting lymphatic vessels draining from the site of PLN removal to the IsLN. These pathways constituted the same drainage pathways as observed at the baseline imaging.

**Figure 7:**
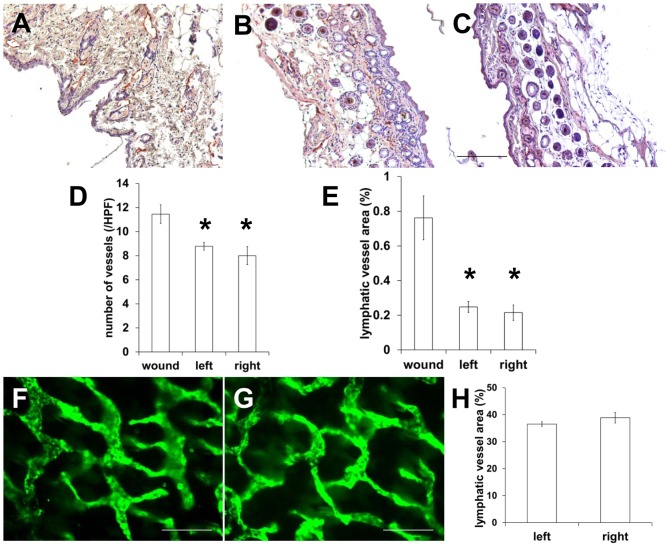
IHC staining for LYVE-1 in skin tissues from (A) the wound region, (B) the left ankle distal to the wound area, and (C) the contralateral right ankle in mice at 2 days post-surgery. Computer-assisted image analysis shows significantly increased number of lymphatic vessels (D) and relative area occupied by lymphatics in the wound area (E), whereas there is no significant difference between tissues from the left ankle in the distal wound site and the contralateral right ankle. Scale: 100 µm. Whole mount staining of lymphatic vessels of (F) the left ankle distal to the wound area, and (G) the contralateral right ankle and (H) fluorescence analysis of vessel area fraction n = 4 mice. Scale bar, 200 µm. * p<0.05 vs. wound.

We observed more fluorescent lymphatic vessels and extravascular ICG around the ankle in the operated hindlimb of mice at day 2 post-surgery. However, it is unknown whether the greater extent of staining of fluorescent lymphatic capillaries is due to distant lymphangiogenesis or is due to backflow in response to resistant fluid dynamics resulting from loss of normal lymph flow pathways. Therefore, we collected tissues from the wound area (red rectangle c in Figure 5) and around the ankle (red rectangle b in Figure 5) distal from the wound site as well as contralateral ankle site for evaluation of lymphatic vessel densities. The percentage of the stained area and the number of the lymphatic vessels were significantly higher in the wound area (Figure 7A) when compared with the tissues in the left ankle distal to wound area (Figure 7B) or contralateral right ankle area (Figure 7C), indicative of active lymphangiogenesis during wound healing. However, we observed no significant difference in the number of lymphatic vessels and the percentage of lymphatic vessel area between the tissues from the left ankle in the distal wound site and the contralateral right ankle as shown in Figure 7D and 7E. This latter result was also confirmed by whole mount staining of lymphatic vessels of the left ankle site and right contralateral ankle (Figure 7F-7H), suggesting that extensive ICG staining of lymphatic capillaries 2 days after PLN surgery results from backflow which is attributable to obstructed normal lymph flow along afferent popliteal lymphatic vessels due to lymphadenectomy.

### B16F10 metastasis after popliteal lymphadenectomy

**Figure 8:**
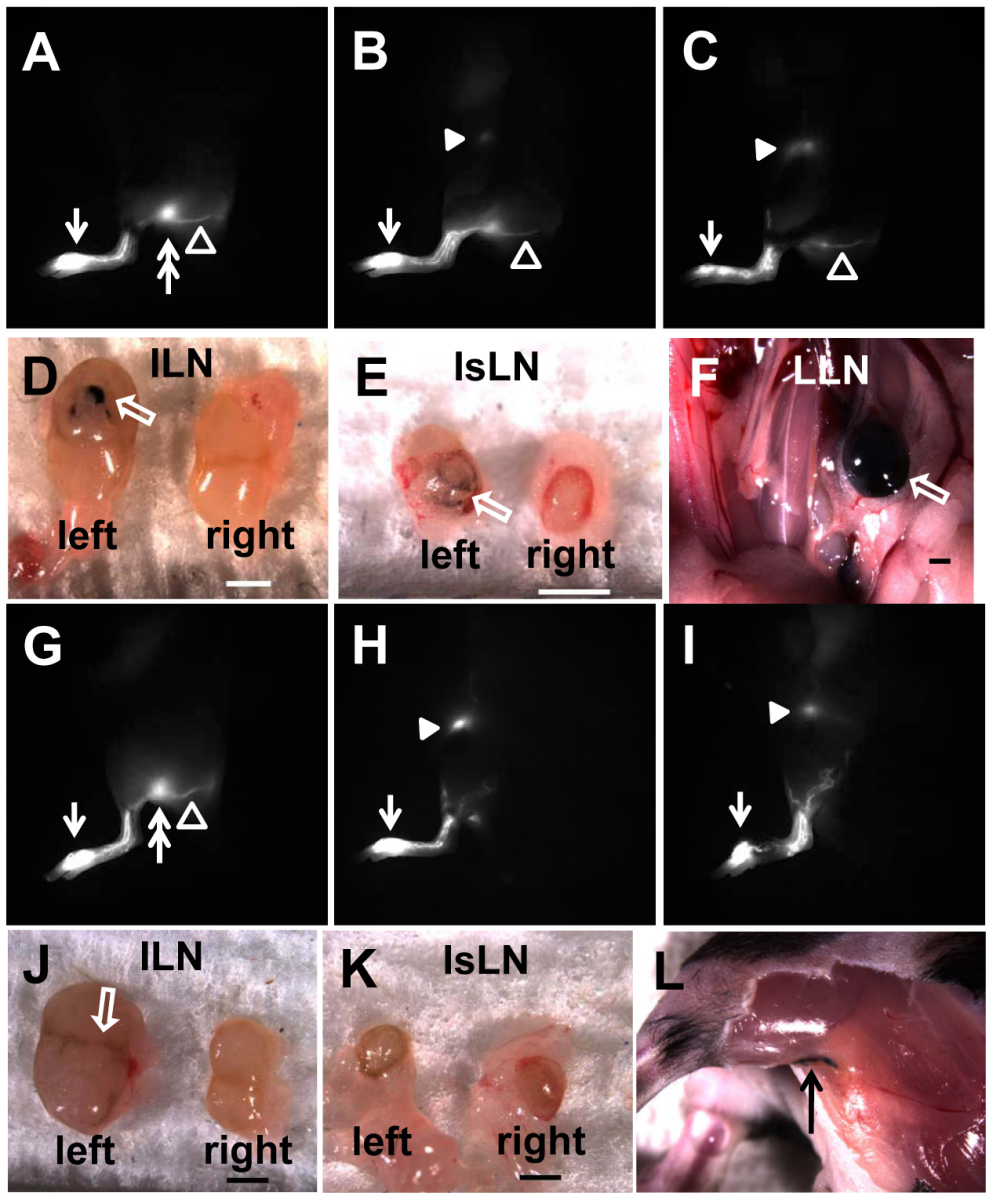
Fluorescent images prior to the popliteal lymphadenectomy (A, G), at 27 days post-surgery (B, H), and 21 days after B16F10 cell inoculation (C, I). At 3 weeks post inoculation, intravital color images showed B16F10 metastasis to the ILN (D), IsLN (E), and LLN (F) in a mouse with lymphatic drainage to ILN and IsLN across the popliteal wound site as demonstrated by the presence of black melanin pigment in LNs. In a mouse with lymphatic drainage to the ILN (I), ILN metastasis (J) was observed, but not in the mouse IsLN which was not involved in the lymphatic drainage pathway (K). In addition, in-transit metastasis was detected proximal to the wound site (L). White arrow, ICG injection site. Double arrow, PLN. Open arrowhead, popliteal efferent collecting lymphatic vessel. Arrowhead, ILN. Open arrow, B16F10 metastasis. Black arrow, in-transit metastasis. Scale bar, 1 mm.

Tumor cells metastasize via lymphatic vessels to the sentinel LN (SLN). It has been shown that murine B16F10 cells inoculated on the paw of mice spread to the tumor-draining PLN. Thus, we sought to investigate where B16F10 cells metastasized to in mice which had previously undergone surgical removal of PLNs. We observed lymphatic drainage to the IsLN and ILN in 3 of 4 mice and to the ILN only in one mouse at 27 days post-surgery. At 3 weeks after B16F10 cell inoculation, intravital imaging data demonstrated that all 4 mice showed LN metastases. Three mice with lymphatic drainage to both the ILN and IsLN through the popliteal wound site showed B16F10 metastases to the ILN and the LLN through the IsLN as shown in Figures 8A-F. One mouse that showed lymphatic drainage only to the ILN represented the ILN metastasis (Figures 8G-F). In addition, we observed in transit metastasis proximal to the surgical area in the mouse (Figure 8L). However, mice without lymphadenectomy showed only PLN metastasis in 2 out of 4 mice (data not shown). Therefore, our data suggests that the popliteal lymphadenectomy results in changes of lymphatic drainage pathways as shown by NIRF lymphatic imaging and thus a pathway for tumor cell dissemination.

### Altered lymphatic function after PLN removal

**Figure 9:**
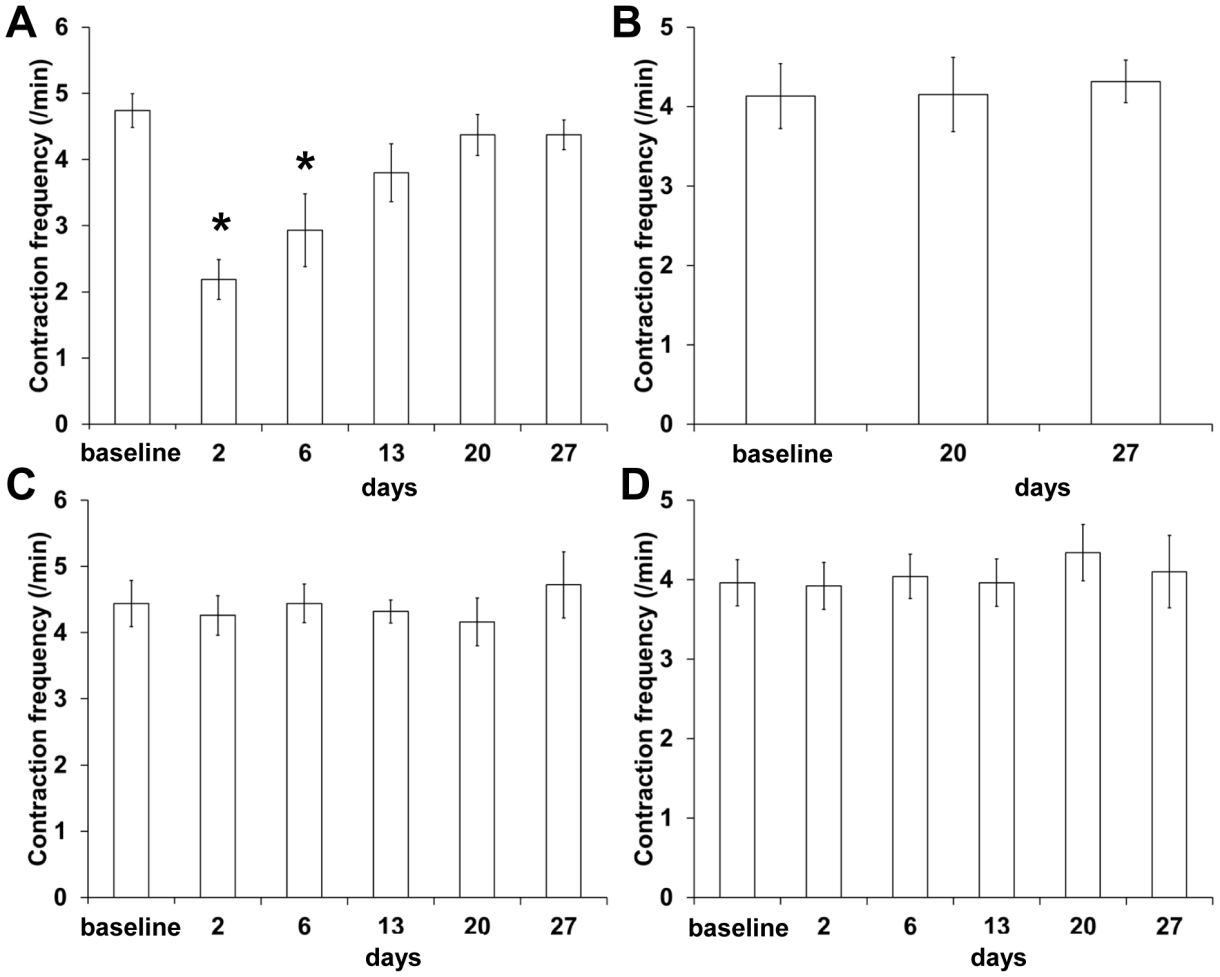
The quantification of lymphatic contractility in the popliteal afferent (A, C) and efferent (B, D) lymphatic vessels in mice after PLN removal (A; n = 19, B; n = 15) and in control mice (C, D; n = 10). * p<0.05 vs. baseline.

As demonstrated above, we longitudinally imaged changes of lymphatic drainage pathways after PLN removal. Because afferent popliteal lymphatic vessels (in the red rectangle c in a baseline white light image in Figure 3) were always observed to drain ICG-laden lymph from the injection site to the PLN prior to surgery and to the site of the PLN removal and/or to the ILN after surgery, we sought to investigate whether lymphatic function in this pathway changed over time after PLN surgery. As shown in Figure 9A, significantly reduced lymphatic contraction frequency was observed in the afferent lymphatic vessels in mice at 2 and 6 days after surgery as compared to the baseline data and control mice (Figure 9C; p<0.05). However, the contraction frequency restored over time, and there was no significant difference in the afferent contraction frequency 13 days after surgery (Figure 9A). Internodal collecting lymphatic vessels connecting from the PLN to the IsLN were observed in a mouse as early as two days after surgery and in 15 of 19 mice from 20 days after post-surgery. In quantifying lymphatic contraction frequency, we observed similar contraction frequency in the vessels between before and 20, and 27 days after surgery (Figure 9B), indicating that the collecting vessels were functionally contractile after lymphadenectomy. Longitudinal imaging with i.d. injection of ICG to control mice did not affect the afferent nor efferent lymphatic contractile function (Figures 9C and 9D, respectively).

## Discussion

In this study, we demonstrate transient changes of lymphatic function and drainage patterns in mice for a period of up to 4 weeks following PLN removal. Since LNs are connected to afferent (higher pressure) and efferent (lower pressure) vessels, it is expected that removal of a LN can cause an increase in intralymphatic pressure. Our data shows a two-step process of recovery from PLN removal. Although minimally invasive PLN removal resulted in less traumatic lymphatic injury and thus mild swelling of the operated hind leg, we observed a complete blockage of normal lymphatic drainage to the popliteal region and thus triggering alternate, pre-existing collateral lymphatic vessels to drain stagnant lymph to the ILN in mice 2 days after lymphadenectomy. We also observed retrograde flow and more fluorescent lymphatic capillaries resulting from a greater extent of dermal backflow on the paw distal from the wound area at day 2. This lymphatic phenotype and alternate drainage pathways may be due to increased resistance to flow as a result of obstructed lymphatics, but not lymphangiogenesis, since there was no significant difference in the number of lymphatic vessels and the percentage of vessel area between tissues collected distally from operated and non-operated limbs (Figure 7). Once lymphangiogenesis resulting in the growth of new functional lymphatic vessels took place and lymphatic continuity and deficient lymph “pumping” was restored, dermal backflow, lymph flow in the retrograde direction in conducting lymphatic vessels, or significant distribution of diffused ICG disappeared due to reduced resistance to flow along the redirected or reconnected drainage pathways. As compared to baseline, three different lymphatic drainage patterns remained at 4 weeks post-surgery: (i) collateral lymphatic vessels draining from the paw to the ILN (Figure 3), (ii) reconnected afferent and efferent popliteal lymphatic vessels across the popliteal wound site and thus lymphatic vessels draining to the IsLN (Figure 5), or (iii) both (Figure 4).

Collateral lymphatic circulation is a common manifestation of lymphatic obstruction and the lymphatic system can use it to overcome pathways rendered nonfunctional due to damage to the lymphatics [31], [32]. Collateral flow patterns are dynamic and difficult to predict, since they depend upon the extent and location of the lymphatic obstruction; however, NIRF imaging data successfully demonstrated different collateral lymph flow patterns due to disruption of normal lymphatic flow dynamics. Collateral lymphatic vessels were observed in all 16 mice only upon removal of the PLN, which had not been observed prior to surgery, and were no longer visualized in 4 of 19 mice at 27 days post-surgery, indicative of recovery of lymphatic drainage along reconnected collecting lymphatics across the popliteal wound site. This alternate lymphatic drainage pathway became detectable due to redirection of lymph flow following PLN removal, since baseline imaging data showed lymphatic drainage to the ILN via collateral lymphatic vessels and to the PLN via popliteal afferent collecting vessels in 3 of 19 normal mice prior to surgery. The observed baseline patterns draining to the ILN in the hind limb of three mice were visualized at 2 days after surgical removal of the PLN, whereas neither lymphatic drainage across the site of LN removal nor additional collateral vessels were detected, further supporting that observed collateral vessels were pre-existing and provided sufficient transport capability during an obstruction of lymphatics. Collateral vessels were still visualized even after reconnection of pre- and post-nodal lymphatic vessels was detected for a period of approximately 4 weeks after surgery. Although resistance to lymph flow changes over time, collateral lymph flow patterns may be preferred for effective lymph transport [33].

The popliteal afferent and efferent collecting lymphatic vessels are well defined and easily visualized *in vivo* with injection of ICG on the dorsal aspect of the paw. Thus, lymphatic function and drainage patterns can be longitudinally characterized in response to minimally invasive PLN dissection, which would affect all lymph flow from the lower hind limb and thus make this model relevant to post-surgical lymphedema. Previously, Ikomi *et al*. [18] demonstrated recanalization of collecting lymphatic vessels after surgical removal of the PLN in rabbits using x-ray following cannulation of a peripheral lymphatic vessel and injection of radiographic contrast material. They showed only popliteal afferent lymphatic vessels and accumulation of the contrast medium at the popliteal region one week after PLN removal; however, at 4 weeks post-surgery, the connection of popliteal afferent and efferent lymphatic vessels, together with collateral lymphatic vessels, were observed [18]. The same group also showed spontaneous recanalization of collecting lymphatic vessels after a minimal excision (1 mm in rabbit and 0.5 mm in mice) of a popliteal afferent lymphatic vessel [18], [34], suggesting collecting lymphatic vessels, similar to initial lymphatic capillaries, can be reconstructed in adult tissues [20]. Interestingly, although the popliteal LN in rabbits over 10 mm in length was surgically excised, reconnected afferent and efferent lymphatic vessels were observed in rabbits; however, no recanalization was observed after excision of 3 mm segment of an afferent collecting lymphatic vessel [18].

Changes of lymphatic drainage patterns have been reported in mice after LN removal, alone or with the associated fat pad containing pre- and post-nodal collecting lymphatic vessels [19], [20], [22], [23], [29]. Unlike the former (similar to our minimally invasive PLN dissection), the latter resulted in severe lymphatic injury and significant increase in limb volume; however, natural resolution of acute lymphedema was observed within 4 weeks after surgery [23], [29]. Mendez *et al*. [23] showed ICG fluorescence throughout the entire forelimb immediately after the axillary LN (ALN) removal due to interstitial spreading of ICG dye, less diffused drainage patterns at day 10, and new functional fluorescent lymphatic vessels draining ICG on the paw, bypassing the surgical wound at day 15. *In situ* imaging after injection of FITC dextran or other visibly excited fluorescent dyes has also been used to image functional maturation of newly formed lymphatic vessels in mice after LN removal and LN transplantation together with *Vegf-c/d* gene therapy [20]. In past studies, lymphatic transport capability was investigated; however, none of the studies have shown changes in lymphatic contractile function over time. We observed significant reduction of lymphatic contraction frequency at day 2 post surgery, with gradual restoration of function over 4 weeks post-surgery. Our data demonstrates transient changes in lymphatic function in mice after surgical removal of the PLN, mainly due to a recovery of a lymphatic drainage deficit.

Since most cancers metastasize through lymphatic routes before spreading to distant organs, regional LN staging is important for prognosis estimation and therapy stratification. ALN dissection (ALND) and pelvic LND (PLND) have been employed for regional control of disease in order to achieve potential cure of breast, prostate, bladder, and other cancers. Our results, employing the metastatic B16F10 model, suggest that co-opting of collateral lymphatic vasculatures and re-direction of lymphatic drainage may negate the use of prophylactic lymphadenectomies to disrupt metastatic pathways.

In breast cancer survivors, ALND is associated with a significant morbidity, with up to 60% of patients who undergo both ALND and radiation therapy suffering at some time from lymphedema [35]. As a result, less invasive SLND is currently employed whereby the first SLN to drain the diseased region is resected and, if proven cancer-positive, then the nodes in the primary draining basins are additionally resected to provide a more accurate prognostic evaluation. However, after SLND alone, lymphedema can still develop and remains a major concern among breast cancer survivors, although the incidence is significantly lower than patients with ALND [36]. Previously, our group and others imaged a lymphatic phenotype in patients with cancer-related lymphedema and showed significant architectural and functional changes which include reduction of functional lymphatic pumping, lymphatic reflux, tortuous and leaky lymphatic vasculature, and inefficient ICG uptake as compared to normal healthy controls [37]–[39]. However, the etiology and developmental stages of this phenotype remains to be assessed with longitudinal imaging to uncover why some, but not all cancer survivors, develop post-surgical lymphedema.

In conclusion, we have non-invasively imaged dynamic lymphatic response to surgical removal of a PLN using NIRF imaging and provided assessment of its impact on metastatic spread. Changes in lymphatic function and drainage patterns via collateral lymphatic vessels were observed for a period of approximately 4 weeks after surgery. We observed dermal backflow, diffused dye patterns, and retrograde flow by day 2 post-surgery, during which lymphatic function was also significantly reduced. However, when lymphatic drainage capability was restored, as evidenced by lymphatic contractility, abnormal lymphatic drainage patterns regressed. Collateral lymph flow patterns to facilitate obstructed lymphatics disappeared in some mice, but were still observed in a majority of mice. These collateral draining pathways, while responsible for surgical repair, can also provide a route for metastatic dissemination of disease.
